# Observation of Transition from Rate Law to Butler–Volmer Controlled Water Oxidation Kinetics on Hematite Photoanodes

**DOI:** 10.1021/jacs.5c18734

**Published:** 2026-01-29

**Authors:** Tianhao He, Daniele Benetti, Cindy Tseng, Benjamin Moss, Detre Teschner, Travis E. Jones, Andreas Kafizas, Michael Grätzel, Simone Piccinin, James R. Durrant

**Affiliations:** ◧ Department of Chemistry, Centre for Processable Electronics, 4615 Imperial College London, London W12 0BZ, U.K.; ‡ Resnik Centre for Sustainability, California Institute of Technology, Los Angeles, California 91125, United States; § Department of Heterogeneous Reactions, Max-Planck-Institute for Chemical Energy Conversion, Mülheim an der Ruhr 45470, Germany; ∥ Department of Inorganic Chemistry, 28259 Fritz-Haber-Institute of the Max-Planck-Society, Berlin 14195, Germany; ⊥ Theoretical Division, 5112 Los Alamos National Laboratory, Los Alamos, New Mexico 87545, United States; # Institut des Sciences et Ingenierie Chimiques, 155550 Ecole Polytechnique Fédéral de Lausanne, Lausanne CH-1015, Switzerland; □ 9327 Consiglio Nazionale delle Ricerche, Istituto Officina dei Materiali, Trieste 34149, Italy; ○ Department of Chemistry, University of Oxford, Oxford OX1 3TA, U.K.

## Abstract

Despite its central role in photoelectrochemical (PEC) water splitting, the mechanistic pathway of water oxidation on metal oxides remains unresolved, with population-based and Butler–Volmer (BV) models offering distinct views on how surface valence band holes drive the reaction. Here, we bring together these two perspectives by combining operando photoinduced absorption (PIA) spectroscopy with photocurrent analyses on α-Fe_2_O_3_ (hematite) photoanodes as a function of light intensity. We find a crossover from population-controlled, rate law water oxidation at low hole densities to a BV-like, potential driven regime at high densities, triggered by band edge unpinning once surface M–OH species are fully oxidized, and excess holes accumulate without compensation. This mechanistic transition unifies competing models of interfacial charge transfer and reveals design principles for optimizing water oxidation in metal oxide photoelectrodes.

## Graphical abstract

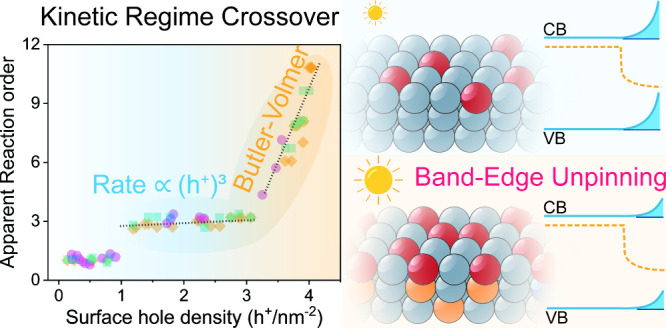

Water oxidation is the key kinetic bottleneck in photoelectrochemical (PEC) and electrochemical (EC) water splitting. Understanding the factors governing its kinetics is critical for improving catalyst performance and enabling efficient green hydrogen production.^1^ In both EC and PEC systems, reaction kinetics are often interpreted using the Butler–Volmer (BV) formalism, in which the rate depends exponentially on the applied potential, reflecting a potential-driven modulation of the activation energy for the rate-determining step (RDS).^2–5^ Classical models by Gärtner and Gerischer extended BV kinetics to semiconductors by incorporating band bending and carrier accumulation, providing a basis for potential-driven charge transfer in PEC systems.^6,7^ These frameworks also account for the additional photovoltage generated under illumination, reflected in the hole quasi-Fermi level during oxidation.^6–12^ More recently, we, and others, have proposed that for water oxidation on metal oxide photoelectrodes, the reaction rate is governed not by potential but by the density of accumulated oxidizing species (e.g., valence band holes or oxidized metal centers). This ‘population model’ results in rate law dependencies of reaction rate on surface hole density, with apparent reaction orders ranging from one to three.^13–19^ Experimental and theoretical support for population-driven water oxidation reaction kinetics has also been reported for the electrocatalyst iridium oxide.^20,21^ As such, a key challenge is to elucidate the factors determining the validity of potential versus population (i.e.: BV versus rate law) reaction kinetics for PEC and EC water oxidation.

The observation of rate law reaction kinetics implies that the reactive species (i.e.: surface holes accumulating at the valence band edge) are energetically equivalent, and that the valence band edge remains pinned regardless of hole accumulation. This contrasts with many studies of PEC systems where band-edge unpinning is observed under irradiation due to surface charge accumulation, leading to BV-type kinetics.^22–27^ For metal oxide photoanodes, it has been proposed that surface hole formation is coupled to proton release, resulting in surface holes being electrostatically neutral (analogous to Mn clusters in Photosystem II^28^), thereby enabling the accumulation of oxidizing species without band-edge unpinning.^14,18^ This is potentially advantageous, as band-edge unpinning can reduce the space charge layer band bending, limiting charge separation.^29^ As such, consideration of rate law versus BV-type kinetics depends critically on the net charge of accumulating species and their impact on the band energetics.

In this study, we use operando photoinduced absorption (PIA) spectroscopy and photocurrent analysis to probe water-oxidation kinetics on hematite across a broad illumination range. We reveal a clear mechanistic crossover: population-controlled, third-order behavior at low light intensities transitioning to a potential-driven, BV-like regime above ∼ 1.1 Sun. Our results show that both kinetic models can describe the same photoanode but under different conditions, governed by whether accumulated surface holes remain proton-compensated (neutral) or become uncompensated (charged).

Nanostructured α-Fe_2_O_3_ photoanodes were grown on FTO substrates by APCVD, yielding nanostructured cauliflower morphology with high surface area.^30^ Under chopped 1 Sun illumination, these photoanodes exhibited photocurrent densities of ∼ 1.8 mA/cm^2^ at 1.23 V_RHE_ (Figure S1), in line with prior literature.^30^ PIA and photocurrent measurements were undertaken under standard PEC conditions in a three-electrode cell at an applied voltage of 1.5 V_RHE_, employing 5 s duration pulsed LED excitation (see SI for details). This bias was chosen to suppress back-electron recombination, as established in our previous work on hematite photoanodes,^14,31^ consistent with the absence of a negative photocurrent spike following light-off (Figure S1). As shown in Figure 1a (and S1–S3 for TiO_2_), PIA signals assigned to surface holes increase with light intensity, with surface hole accumulation approaching saturation at high flux.^14^ Probe light at 650 nm was used to track these surface holes, assigned primarily to surface Fe­(IV)=O species.^14,19,32,33^ Corresponding transient photocurrent traces (Figure 1b) confirm efficient charge extraction and minimal back-electron recombination under these conditions as highlighted by the absence of negative spikes on light turn off.

**Figure 1:**
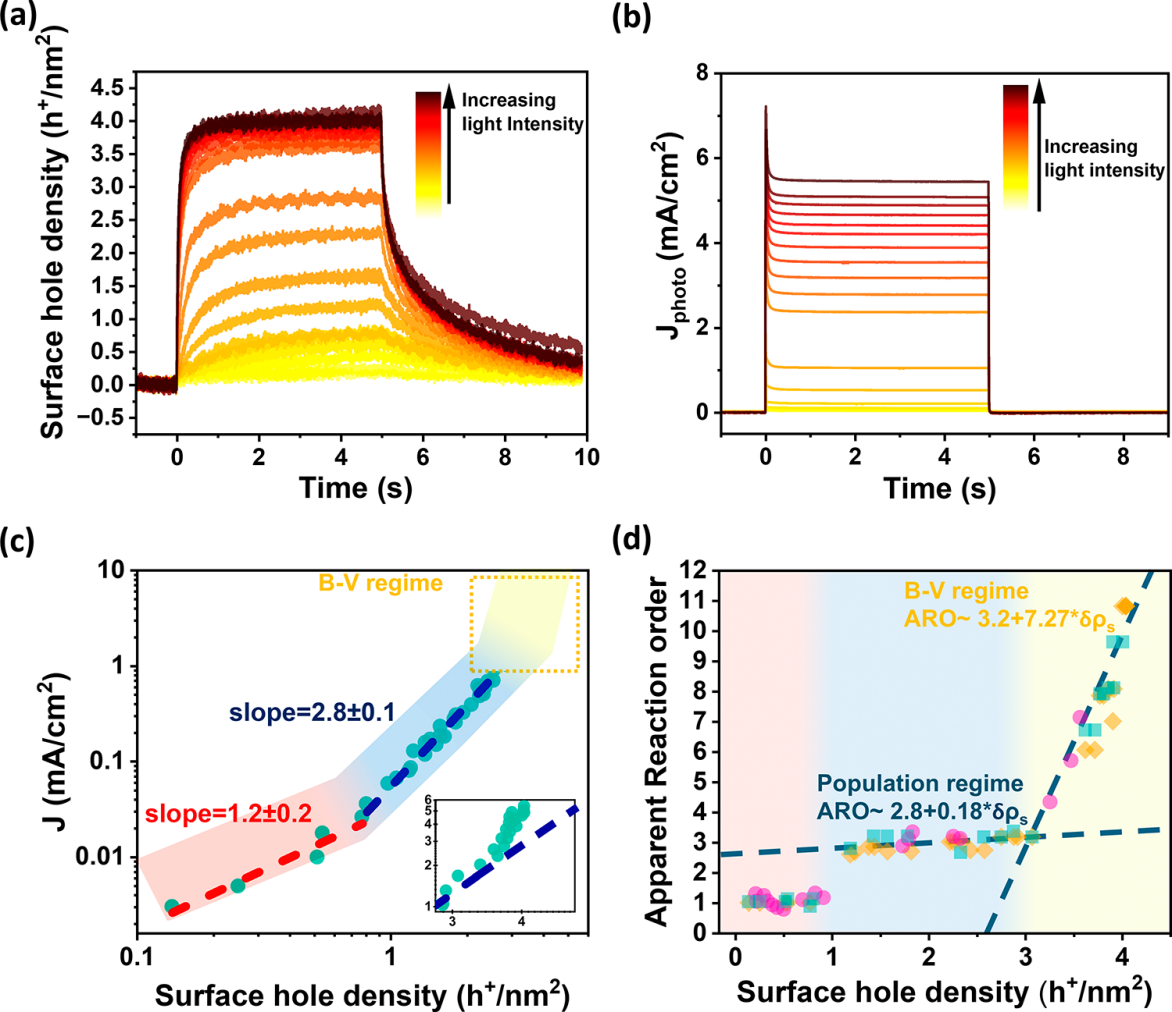
(a) PIA and (b) TPC measurements of Fe_2_O_3_ in 1 M NaOH at 1.5 V_RHE_, probed at 650 nm and excited with a 365 nm LED light (5 s pulses, 0.05 ÷ 49 mW cm^–2^), (c) log–log plot of quasi-steady-state hole density (from PIA) vs TPC; inset: zoom-in of boxed region. (d) Reaction order vs hole density extracted by three methods from panel c (see SI).

Apparent reaction orders (ARO) were extracted from PIA and photocurrent data using established methods (see SI).^14^ In the framework of the population-controlled rate law model, the ARO is obtained from the plot of J versus surface hole density (*p*_s_) on a log–log scale, and is defined as

$$\mathrm{ARO}=\frac{d\log\left(J\right)}{d\log\left(p_{s}\right)}$$

As shown in Figure 1c, increasing light intensity up to ∼ 2.5 Sun (corresponding to ∼ 4 h^+^/nm^2^) results in higher surface hole densities and photocurrents. Initially, the ARO transitions from first to third order, consistent with previously reported rate-law behavior.^13,14,18^ Strikingly, above ∼ 1.1 Sun, the photocurrent begins increasing more steeply with hole density (inset, Figure 1c), and the ARO rises sharply, indicating a breakdown of the population model at high irradiance. To validate this kinetic crossover, we applied three independent methods to extract the ARO from PIA data: current density vs hole density, initial PIA decay kinetics, and decay slope analysis (Figure 1d and S3b; see SI). All three methods consistently show a constant ARO≈3 between 1–3 h^+^/nm^2^, followed by a marked increase at higher densities. This confirms a transition at ∼ 3 h^+^/nm^2^ from a population-controlled regime to a distinct regime where ARO increases with hole density. This deviation at high light intensities is consistent with recent reports using alternative methods, such as voltage-induced absorption (VIA), which also reveal kinetic anomalies on hematite under strong illumination.^34,35^ To test the generality of the observed breakdown, we conducted analogous measurements on dense, flat anatase TiO_2_ photoanodes. As shown in Figure S3, these samples also exhibit a transition from second-order kinetics up to ∼ 2.5 h^+^/nm^2^ to a regime where the reaction order increases sharply with hole density. Together, these findings suggest that the breakdown of population-controlled kinetics is a general feature of metal oxide photoanodes and not limited to a specific material or measurement technique.

Close inspection of Figure 1d shows that the ARO increases slightly with surface hole density in the population-controlled regime (∼ 1 < p_s_< ∼ 3 h^+^/nm^2^), fitting well to ARO = 2.8 + 0.18 δ*p*_s_. Here, δp_s_ refers to the change in surface hole density relative to the start of the regime, i.e., δp_s_ = p_s_ – p_s_0, where p_s_0 is the threshold hole density at the onset of the regime. This weak dependence on surface hole density agrees well with our recent microkinetic model for PEC water oxidation on hematite based on DFT calculations.^19^ This model predicts a RDS involving a 3-hole elementary step leading to the formation of a superoxo intermediate, which displays a weak dependence of its activation energy on the surface hole density. Our DFT/microkinetic modeling calculated that this step has an activation energy *E*_a_ = 0.16 eV - 0.0073 *p*_s_, resulting in ARO = 3.07 + 0.29 δ*p*_*s*_, in good agreement with our experimental measurements (see also SI, section 2, for details on the model and the derivation of the ARO). However, once the surface hole density exceeds ∼ 3 h^+^/nm^2^, the experimental measurements indicate that the ARO rises steeply, exhibiting a linear dependence on surface density of the form ARO ≈ 3.2 + 7.27 δ*p*_s_. This steep gradient in ARO with surface hole density cannot be explained by our microkinetic model and suggests that there is a new mechanism determining the ARO at high light intensities.

To ensure that the breakdown of rate-law behavior does not arise from artifacts, we evaluated alternative explanations (see SI for details). Variations in absorption coefficient are unlikely, as transmission-mode PIA probes the full film and surface/subsurface holes exhibit similar spectra. Local heating was ruled out experimentally: a 2.5 Sun illumination test produced only a 0.2 °C rise after 5 min, far exceeding the <10 s illumination used during PIA. Having excluded these effects, we focus on the remaining explanation, the accumulation of uncompensated holes, leading to electrostatic band-edge unpinning and the emergence of BV-type kinetics.

Recently, Bevan and Peter have modeled the impact of band edge unpinning on PEC water oxidation kinetics,^36^ showing that in the limit of no proton release (i.e., when surface holes are not locally charge compensated) electrostatic repulsion can unpin the band edges, inducing a potential drop across the Helmholtz layer, and giving rise to a BV-like behavior. In this unpinned regime, they predict that the ARO should increase linearly with surface hole density *p*_s_. Their model further indicated that such behavior becomes dominant at high densities of uncompensated (charged) holes, where electrostatic effects significantly alter interfacial energetics. In this scenario, the ARO would scale as^36^

$$\mathrm{ARO}={\mathrm{ARO}}_{0}+\frac{q^{2}\gamma }{k_{B}TC_{H}}\delta p_{s}$$

where ARO_0_ represents the apparent reaction order in the absence of electrostatic effects, *q* is the elementary charge, γ is a unitless proportionality constant related to the Tafel model, *k*_B_ is Boltzmann’s constant, *T* is temperature, *C*_H_ is the Helmholtz layer capacitance, and *δp*_*s*_ is the change in surface hole density. In Bevan and Peter’s model, ARO_0_ = 1 reflects their assumption of simple first-order kinetic.

Using a value for *C*_H_ of 100 μF/cm^2^ and assuming γ = 0.6, as suggested by Bevan and Peter, this model predicts an ARO that increases linearly with respect to surface hole density with a gradient of 3.8 (h^+^/nm^2^)^−1^. Employing our first-principles estimate for *C*_H_ of 40–50 μF/cm^2^, depending on surface termination,^37^ we obtain 7.7–9.6 (h^+^/nm^2^)^−1^. Strikingly, the predicted range aligns closely with our experimentally observed gradient of 7.3 (h^+^/nm^2^)^−1^ at high illumination. This agreement supports the idea that the breakdown of rate law behavior above 3 h^+^/nm^2^ could indeed result from the onset of band edge unpinning, and thus an acceleration in water oxidation kinetics due to an increasing potential drop across the Helmholtz layer, typically referred to as Butler–Volmer behavior. While our microkinetic model assumes surface charge neutrality and cannot capture this transition,^19^ a full quantitative treatment would require modeling the shift in activation energy as a function of capacitive charging, an important, yet complex, task beyond the scope of this work.

The analysis above reveals two regimes of water oxidation kinetics on hematite photoanodes. At low light intensities, surface holes are effectively charge-compensated via proton release, consistent with rate-law behavior and band edge pinning. Above ∼ 1.1 Sun, this compensation mechanism becomes insufficient, leading to the accumulation of uncompensated holes. This results in band-edge unpinning, where the reaction rate becomes sensitive to the hole quasi-Fermi level, in line with Butler–Volmer-type kinetics. Additional evidence for band unpinning comes from the photocurrent response (Figure 2): below 1.1 Sun, the photocurrent scales linearly with light intensity, indicating efficient charge separation and pinned band edges. In contrast, beyond 1.1 Sun, the photocurrent increases sublinearly, suggesting suppressed band bending and reduced separation efficiency. This shift correlates with the onset of a sharp increase in the ARO, reinforcing the link between a change in interfacial electrostatics and the kinetic transition.

**Figure 2:**
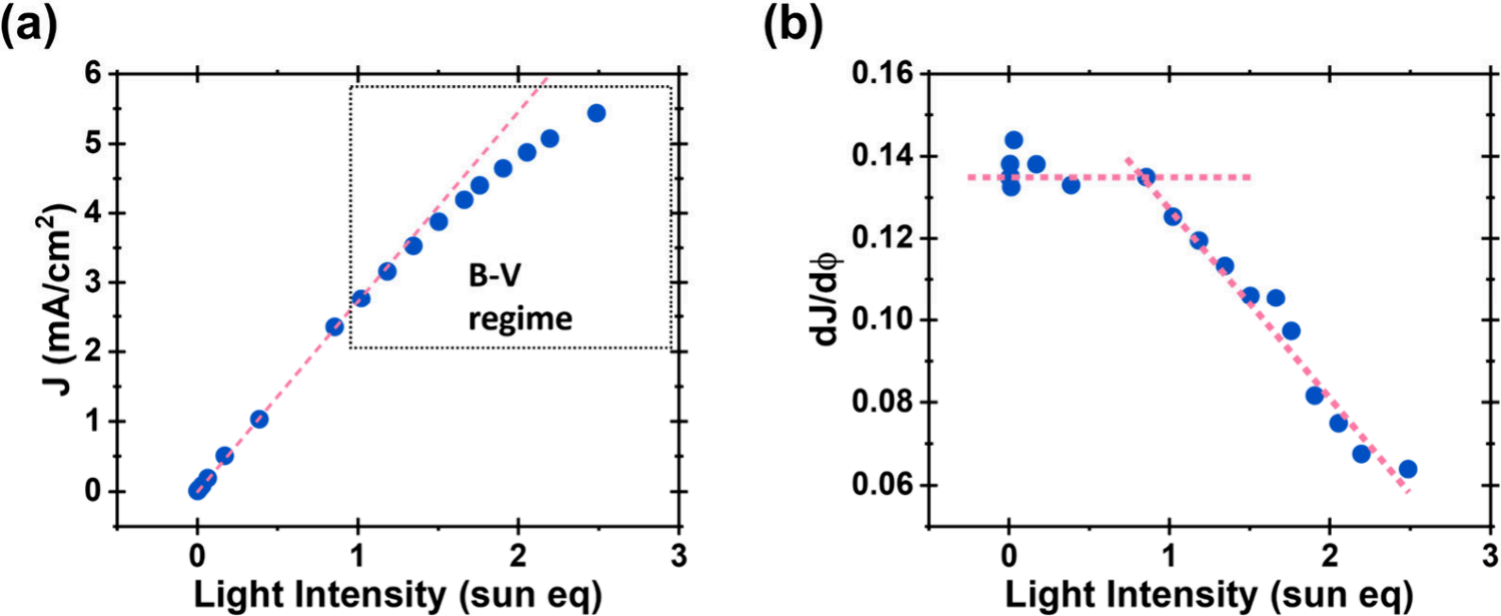
(a) Photocurrent vs light intensity for α-Fe_2_O_3_ at 1.5 V_RHE_. (b) First derivative of photocurrent with respect to light intensity (Φ), as a proxy for charge separation efficiency.

Below this value, charge compensation likely occurs through the oxidation of surface Fe–OH to Fe=O, accompanied by proton release (the high OH^–^ concentration in 1 M NaOH ensures efficient buffering, minimizing local pH effects, however a minor contribution from OH^–^ adsorption cannot be excluded).^38^ Our interpretation is further supported by prior estimates of surface hydroxyl group densities on hematite (2–6 nm^–2^),^39,40^ in alignment with the threshold we observe. Once these sites are saturated, further hole accumulation results in uncompensated positive charge, driving band-edge unpinning. These excess holes may localize on subsurface sites or regions devoid of −OH groups. In comparison, TiO_2_ exhibits an earlier transition (∼2.5 h^+^/nm^2^), likely due to its lower surface −OH density and acidity (p*K*_a_), which could lead to earlier saturation of proton-compensated sites.

Overall, our findings reveal a kinetic crossover in photoelectrochemical water oxidation: from a population-controlled, multihole regime to a potential-driven, Butler–Volmer-like regime at high light intensities, as illustrated in Figure 3. This transition arises from surface site saturation, accumulation of uncompensated holes, and the resulting band edge unpinning, which shifts the interfacial potential drop into the Helmholtz layer. As such, we have reconciled two previously competing mechanistic models, showing they can describe distinct operating regimes of the same photoanode, governed by whether surface holes remain proton-compensated. This mechanistic transition is likely general to many metal oxide semiconductors, not limited to TiO_2_ and Fe_2_O_3_, and highlights a key design principle: maintaining proton-coupled, charge-neutral hole accumulation is essential for preserving efficient, rate-law-driven water oxidation.

**Figure 3:**
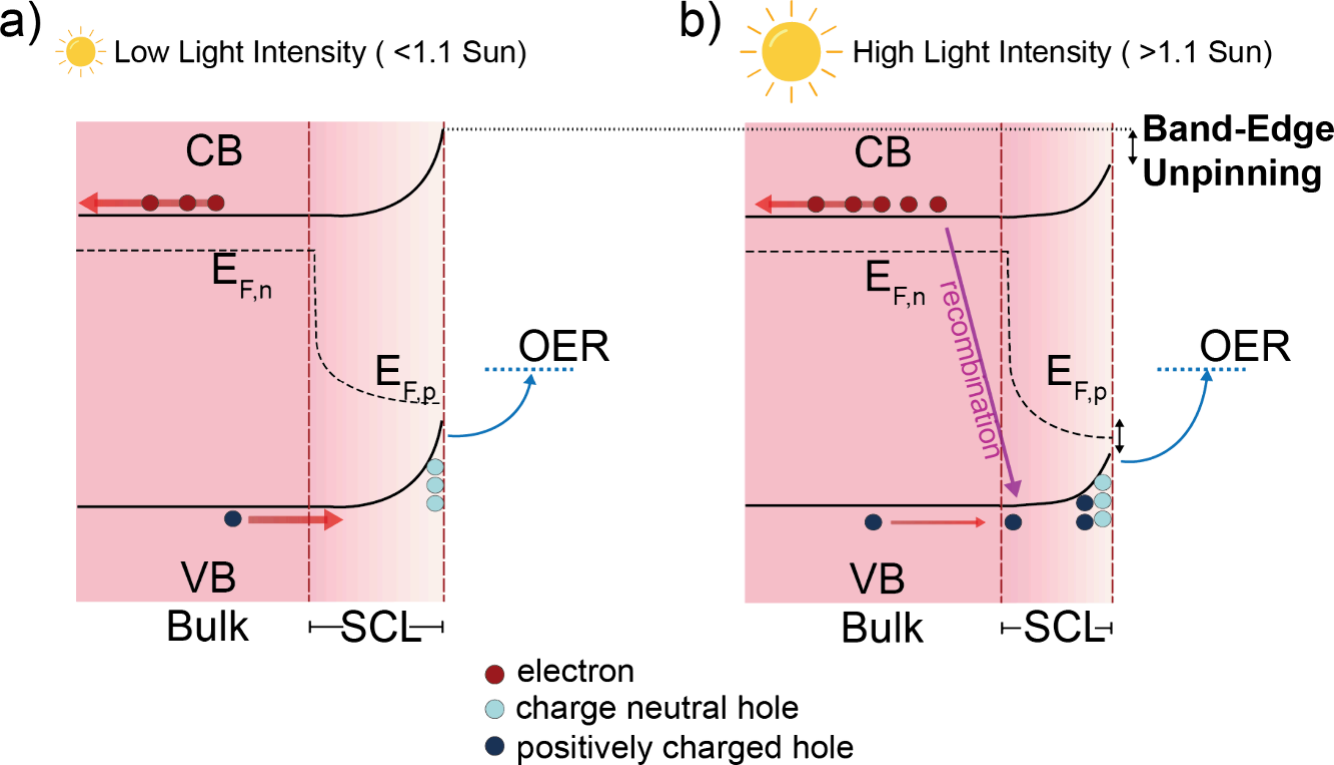
Schematic of the mechanistic transition in hematite photoanodes under increasing light intensity. (a) Under moderate illumination (≤1.1 Sun), photogenerated holes accumulate at the surface and remain charge-neutral (proton-coupled). This maintains band edge pinning and third-order, population-controlled kinetics. (b) At higher light intensities (>1.1 Sun), surface active sites become saturated. Additional holes accumulate as uncompensated charge below the surface, generating a local electric field that shifts the potential drop into the Helmholtz layer (band edge unpinning). The reaction becomes potential-driven, exhibiting Butler–Volmer kinetics and increased interfacial recombination losses.

## Supplementary Material



## Notes

Contains full experimental methods, PIA setup details, data analysis procedures, additional figures (Figures S1–S5), and supporting discussion for reaction order extraction, band edge unpinning analysis, and control experiments on TiO_2_ photoanodes (PDF)
