# Supplementary material for: Observation of Transition from Rate Law to Butler–Volmer Controlled Water Oxidation Kinetics on Hematite Photoanodes
Source: J Am Chem Soc. 2026 Jan 29;148(5):4833–8. doi: 10.1021/jacs.5c18734 (PMC12903865; doi:10.1021/jacs.5c18734)
Supplement: Supplementary file 1 [file ja5c18734_si_001.pdf]

Observation of transition from rate law to Butler-Volmer controlled water oxidation kinetics on Hematite Photoanodes

Tianhao He[1,†], Daniele Benetti[1,†,*], Cindy Tseng[1], Benjamin Moss[1,2], Detre Teschner[3,4], Travis E. Jones[5], Andreas Kafizas[1], Michael Grätzel[6], Simone Piccinin[7,*], James R. Durrant[1,8*]

1. Department of Chemistry, Centre for Processable Electronics, Imperial College London, London W12 0BZ, U.K.

2. Resnik Centre for Sustainability, California Institute of Technology, Los Angeles, California 91125, United States

3. Department of Heterogeneous Reactions, Max-Planck-Institute for Chemical Energy Conversion, Mülheim an der Ruhr 45470, Germany

4. Department of Inorganic Chemistry, Fritz-Haber-Institute of the Max-Planck-Society, Berlin 14195, Germany

5. Theoretical Division, Los Alamos National Laboratory, Los Alamos, New Mexico 87545, United States

6. *Institut des Sciences et Ingenierie Chimiques, Ecole Polytechnique Fédéral de Lausanne, CH-1015 Lausanne, Switzerland*

7. Consiglio Nazionale delle Ricerche, Istituto Officina dei Materiali, Trieste, Italy

8. Department of Chemistry, University of Oxford, Oxford OX1 3TA, U.K.

† These two authors contributed equally to this work

** Corresponding authors*

SUPPLEMENTARY INFORMATION

Contents

SECTION 1: Rate law equation 2

SECTION 2: Discussion of gradient in order-three region 3

SECTION 3: Possible hypothesis for the breakdown of rate law 4

Experimental Section: 5

Figure S1 Current-voltage characterization 6

Figure S2 (a) Time-resolved PIA measurements and (b) TPC measurements of an anatase TiO_2 photoanode 6

Figure S3(a) Logarithmic relationship between quasi steady-state surface hole density (converted from PIA amplitude) and TPC under different light intensities of TiO2 photoanode(b) Reaction order obtained from (a) under different surface hole densities by using three different methods. 6

Figure S4 First derivative analysis of PIA decay for (a) a haematite photoanode and (b) an anatase TiO2 photoanode conducted under low, medium, and high light intensities, corresponding to the first order, third order, and exponential regimes, respectively, in our rate law analysis. The dashed line represents the tangent line of the curve, which is the first derivative of the curve. 7

Figure S5 The initial slope (reaction order) of the first derivative of the PIA signal relative to the PIA signal amplitude is presented at three light intensities for (a) a haematite photoanode and (b) an anatase TiO2 photoanode. The slope during PIA decay of the first derivative of the PIA signal relative to the PIA signal amplitude at one light intensity for (c) α-haematite and (d) anatase TiO2 photoanode. 7

Figure S6 J–V curves of hematite photoanodes recorded under varying illumination intensities (0.4 to 3.0 suns) in 1 M NaOH (pH 14), scan rate: 20mV/s 8

SECTION 1: Rate law equation

$\frac{dp_s}{dt} = J_{holes} - k_{WO} \cdot p_S^{\beta}$ $(eq\ S1)$

Where p_s is surface hole densities, J_{holes} represents the flux of holes towards the surface, k_{WO} is the water oxidation constant and β is the reaction order. Under steady state conditions, the amount of surface holes accumulated on the surface is not changing, which means $\frac{dp_s}{dt}$ is zero, and the photocurrent, which is the flux of electrons exiting the system, is equal to the flux of holes exiting the surface. The equation under steady state conditions can be rewritten as follows (equation 2):

$photocurrent = J_{holes} = k_{WO} \cdot p_S^{\beta}$ $(eq\ S2)$

From equation S2, reaction order β can be obtained by calculating the slope of a log-log plot of the quasi steady-state photocurrent and surface hole density measured at different light intensities.

Decay rate slope and initial rate slope methods:

With the LED off, the photocurrent drops to zero, implying that J_{holes} also becomes zero. Consequently, Equation S1 is modified as follows (Equation S3):

$\frac{dp_s}{dt} = -k_{WO} \cdot p_S^{\beta}$ $(eq\ S3)$

As shown in Figure S4, the derivative of the PIA decay $(\frac{dp_s}{dt})$ varies at different surface hole densities. Specifically, at high surface hole densities, the derivative assumes a higher value, whereas at reduced surface hole densities, the derivative decreases correspondingly. This relationship underlines the direct correlation between the rate of change in the PIA signal decay and the density of photo-induced holes at the surface. The chopped current-voltage curves (Figure S1) show that back electron-hole recombination is effectively suppressed under the strong anodic bias conditions of these PIA measurements, with the slow decay of the PIA signal when the LED is switched off corresponding to the slow water oxidation process that occurs from the photoinduced holes that remain on the surface. Therefore, the reaction order of the water oxidation reaction can be determined by analysing the relationship between the derivative of the PIA signal and the PIA signal amplitude, as depicted by a linear relationship on a log-log plot, in accordance with Equation S3. The first method for analysing the reaction order, based on Equation S3, involves calculating the initial decay derivative of PIA across different regimes.

Figure S5 (a) and (b) illustrate the initial decay analysis of three PIA decays from three regimes. Employing the same equation S3, the second method entails analysing the derivative during the entire recovery process of a single PIA decay under high light intensity, as depicted in Figure S5 (c) and (d). It is evident from these two kinds of analysis that the reaction order approximates to first order at low surface hole densities (less than 1 h^+/nm^2), transitions to third order behaviour at medium surface hole densities (ranging from 1 to 3

h^+/nm^2), and ultimately exhibits exponential behaviour at high surface hole densities (greater than 3 h^+/nm^2).

SECTION 2: Discussion of gradient in order-three region

To describe the rate law model, we can utilize the microkinetic model developed by some of us that accurately describes the system behaviour within the rate law regime.[1] Here we briefly summarize the main features of the microkinetic model. For more details we refer the reader to the original publication[1]. The model is based on the work by Wang et al. on photoelectrochemical OER on TiO_2.[2] Among the reagents we consider a concentration of photogenerated valence band holes in the space charge layer (SCL), C(h^+), that can oxidize the surface intermediates. Surface holes are assumed to be localized on surface intermediates, e.g. in surface oxygen atoms ($*O^{1-}$), as suggested by DFT calculations[1]. The model does not describe the generation, recombination and diffusion of holes in the SCL, hence the values of the SCL-hole concentration cannot be compared with an experimental estimate of this quantity. The reactions included in the model are listed in Table 1.

Table 1: List of the elementary steps include in the microkinetic model of Righi et al.[1]

**Reactions**
1: $*OH^- + h^+ + OH^- \rightarrow *O^{1-} + H_2O$
2: $*O^{1-} + h^+ + OH^- \rightarrow *OOH^-$
3: $*OOH^- + h^+ + OH^- \rightarrow *OO^- + H_2O$
4: $*OO^- + h^+ + OH^- \rightarrow O_2 + *OH^-$
5: $*OO^- + *O^{1-} + H_2O \rightarrow O_2 + 2*OH^-$
6: $2*O^{1-} + H_2O \rightarrow *OOH^- + *OH^-$
7: $3*O^{1-} + H_2O \rightarrow *OO^- + 2*OH^-$
8: $2*O^{1-} + OH^- \rightarrow *OOH^- + *O^{2-}$
9: $3*O^{1-} + OH^- \rightarrow *OO^- + *OH^- + *O^{2-}$
10: $*O^{2-} + h^+ \rightarrow *O^{1-}$

The model is solved for steady-state conditions as a function of the concentration of the valence band holes C(h^+), at room temperature and at pH=8. The choice of pH is motivated by the reported experimental point of zero charge (PZC) of hematite, 7.9 ± 1.7.[3] The rate constants for the elementary steps are derived from transition state theory, assuming a standard prefactor of $k_B T/h$. The reaction energies and activation energies have been computed from first-principles, using DFT calculations, considering charge neutral surfaces (PZC conditions). The DFT calculations show that these quantities depend on the surface concentration of holes, in an approximately linear fashion. The microkinetic model incorporates these linear dependences. Since the coverage of the surface holes is both an output of the model and an input necessary to compute the reaction energies and barriers, the model needs to be solved self-consistently.

The model predicts that the O-O bond takes place via a 3-hole step, reaction 9 in Table 1, and the log/log plot of the rate of the OER vs. surface hole coverage displays a linear behaviour (see Fig. 4b in Righi et al.[1]). The reaction rate as a function of the surface hole coverage can therefore be fitted with the following rate law:

$$r_{OER}(p_s) = k_{OER}[p_s]^{\alpha}$$

with $\alpha = 3.07$. Since the barriers of the model depend linearly on the surface hole concentration, we can express the rate constant k_{OER} as:

$$k_{OER} = k_0 e^{-\frac{E_a(p_s)}{k_B T}} = k_0 e^{-\frac{Ap_s + B}{k_B T}}$$

Using the chain rule, we find that the apparent reaction order, ARO, has the following form:

$$ARO = \frac{d\,\log(r_{OER})}{d\,\log\,(p_s)} = \alpha - \frac{A}{k_B T}\delta p_s$$

We define $\delta p_s = p_s - p_{s0}$. Since the reference coverage is zero ($p_{s0} = 0$), $\delta p_s = p_s$. As long as the dependence of the apparent activation energy E_a on p_s can be approximated with that of the activation energy of the RDS – reaction 9 in our model – the ARO will have a very small dependence on the surface hole concentration. This is because reaction 9 has a weak dependence on p_s: $E_a^9(p_s) = 0.16\,eV - 0.0073\,p_s$, resulting in $ARO = 3.07 + 0.29\,\delta p_s$. This is in excellent agreement with the measurements reported in the main text, indicating $ARO = 2.8 + 0.18\,\delta p_s$.

However, the microkinetic model described above cannot describe the BV-like regime. This would require incorporating in the model the effects of band edge unpinning and surface charging on the reaction energies and activation energies.

SECTION 3: Possible hypothesis for the breakdown of rate law

Several hypotheses may explain the deviation from rate-law behaviour observed above ~3 h^+ nm^{-2}. We systematically evaluated these possibilities:

(i) Changes in absorption coefficient of surface holes.

At very high hole densities, trapped holes might in principle exhibit lower molar extinction coefficients, causing PIA to underestimate p_s. This explanation is unlikely because our transmission-geometry PIA measurements probe the full film thickness, and prior simulations and transient absorption studies show that surface and sub-surface holes in hematite possess nearly identical spectral signatures.[1] Thus, differential absorption cannot explain the abrupt kinetic change.

(ii) Local heating under high-intensity illumination.

Temperature could affect the reaction rate or the PIA signal. However, hematite J–V behaviour under anodic bias is known to be only weakly temperature-dependent,[4] and a sharp transition in apparent reaction order (ARO) would not be expected from modest temperature increases. To test this, we illuminated the sample continuously at 2.5 suns for 5 minutes. The measured temperature rise was only 0.2 °C, within the error of our IR thermometer. Because PIA illumination lasts <10 s per acquisition, actual heating during kinetic measurements is negligible. We therefore exclude temperature-induced artefacts.

(iii) Accumulation of uncompensated surface charge (band-edge unpinning).
At low hole densities, valence-band holes are proton-compensated via oxidation of Fe–OH groups, enabling charge-neutral accumulation. Beyond a critical density (~3 h^+ nm^{-2}), these sites saturate and excess holes accumulate without proton release. Their uncompensated charge generates interfacial electrostatic repulsion, shifting the potential drop into the Helmholtz layer and unpinning the band edges. This behaviour is consistent with the rising ARO, suppressed photocurrent efficiency, and predictions from the Bevan–Peter model.[5]

Given that hypotheses (i) and (ii) cannot explain the observed sharp kinetic transition, and that (iii) is consistent with both theory and experimental signatures, we conclude that band-edge unpinning driven by uncompensated holes is the most plausible origin of the kinetic crossover.

Experimental Section:

Preparation of haematite and TiO_2 photoanodes

The hematite and TiO_2 photoanodes used in this study were synthesized following previously reported APCVD protocols.[6,7] Hematite films exhibit a nanostructured cauliflower morphology with a roughness factor of ~21, while TiO_2 films display a dense, flat morphology with a roughness factor of ~1.

Photo-Induced Absorption (PIA) Spectroscopy:

The PIA setup is similar to the setup we previously reported.[7, 8] The PIA measurements were carried out for a duration of 20 seconds, utilizing pulses from a UV LED light (365 nm) with a cycle of 5 seconds on and 15 seconds off. This setup utilized a Bentham IL1 tungsten lamp as probe, with a specific wavelength of 650 nm chosen through a monochromator. A long pass filter of 620 nm from Comar Instruments was employed to minimise the LED pump light reaching the detector and avoid photoexcitation of the sample. Photon transmission was captured by a Hamamatsu S3071 Si photodiode. The detected signal was logged using a National Instruments (NI USB-6211) DAQ card. A MOSFET (STF8NM50N from STMicroelectronics) and a frequency generator (TG300 from Thurlby Thandar Instruments) controlled the LED pulse. The LED power is provided by a QL564P power supply from TTi company. The program software is operated through the LabVIEW platform, and further data analysis is processed by Origin Lab software. The absorption of continuous probe light (650 nm) by electronic charges activated by a 365 nm LED pulse results in a reduced transmittance through the sample, manifesting as an increase in absorbance. The change in absorbance is caused by the accumulation of photoinduced charge carriers generated by the band-gap excitation of LED. We note that the reference to "~1 sun" irradiation throughout the main text and Supplementary Information corresponds to the photon flux adjusted to match the photocurrent densities typically observed under AM 1.5G conditions.

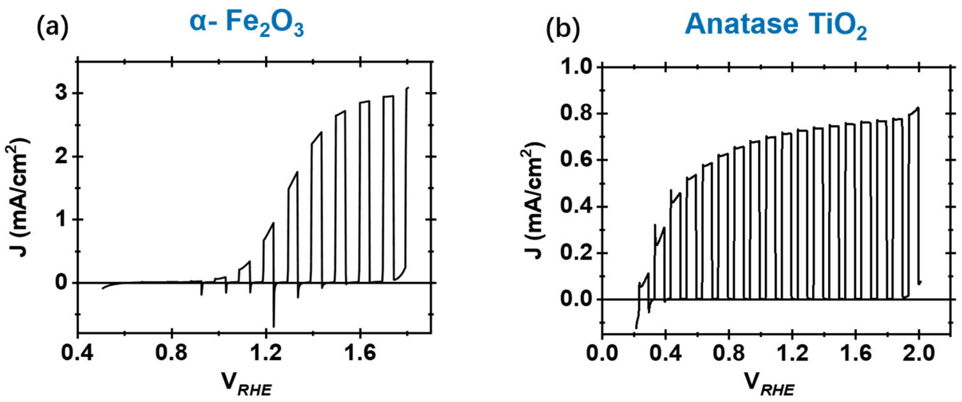

Figure S1 Current-voltage characterization of (a) a haematite photoanode in 1M NaOH solution (pH=14) under 19.7 mW/cm^2 chopped 365 nm LED irradiation with 1.5 V_{RHE} bias and (b) an anatase TiO_2 photoanode in 0.1 M potassium phosphate buffer solution (pH=7) under 8.0 mW/cm^2 chopped 365 nm LED irradiation with 1.5 V_{RHE} bias.

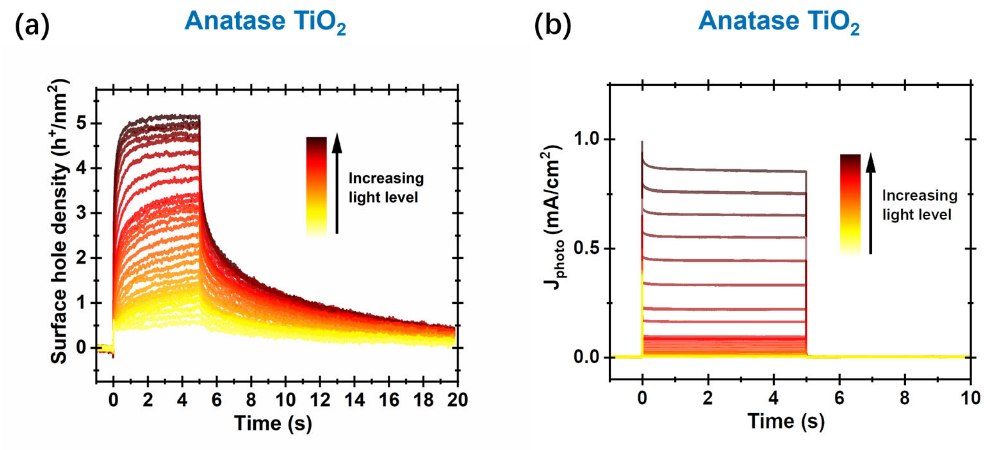

Figure S2 (a) Time-resolved PIA measurements and (b) TPC measurements of an anatase TiO_2 photoanode in 0.1 M potassium phosphate buffer solution (pH=7.2), recorded at 500 nm and excited with a 365 nm LED light for 5 seconds under 1.5 V_{RHE}, light intensities ranging from 0.05 to 22 mW/cm^2.

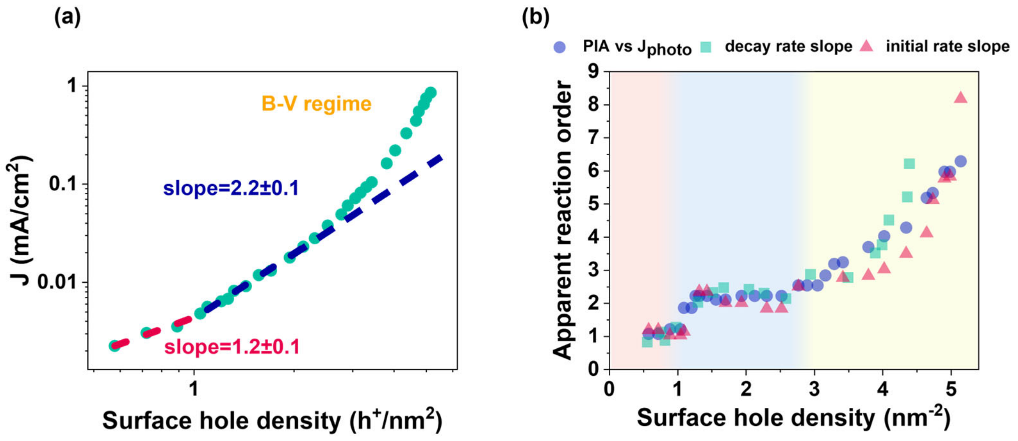

Figure S3 (a) Logarithmic relationship between quasi steady-state surface hole density (converted from PIA amplitude) and TPC under different light intensities of TiO2 photoanode(b) Reaction order obtained from (a) under different surface hole densities by using three different methods.

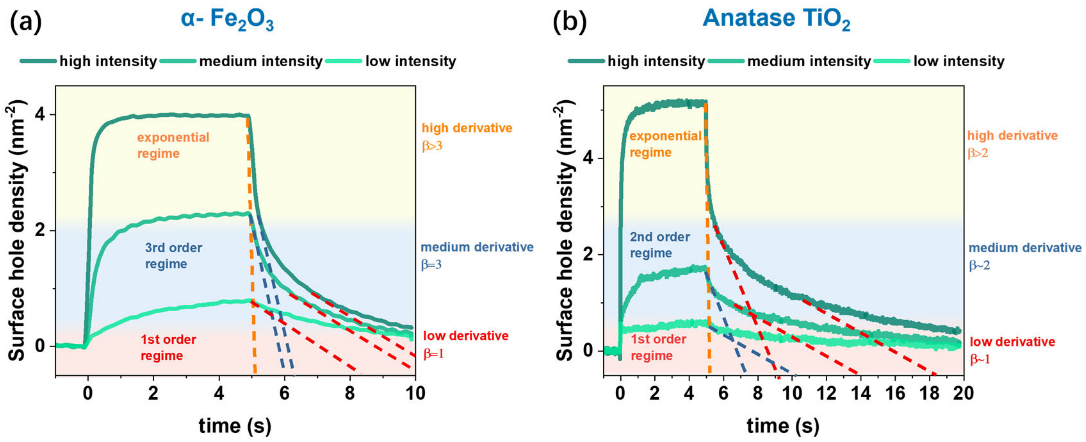

Figure S4 First derivative analysis of PIA decay for (a) a haematite photoanode and (b) an anatase TiO2 photoanode conducted under low, medium, and high light intensities, corresponding to the first order, third order, and exponential regimes, respectively, in our rate law analysis. The dashed line represents the tangent line of the curve, which is the first derivative of the curve.

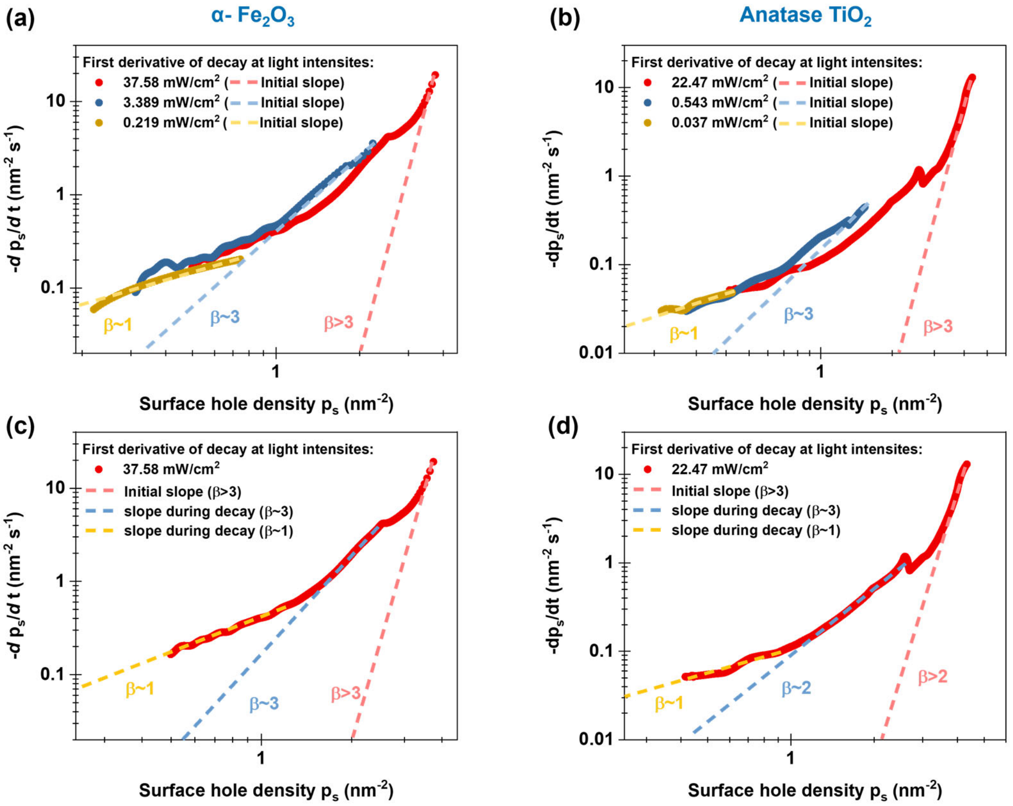

.

Figure S5 The initial slope (reaction order) of the first derivative of the PIA signal relative to the PIA signal amplitude is presented at three light intensities for (a) a haematite photoanode and (b) an anatase TiO2 photoanode. The slope during PIA decay of the first derivative of the PIA signal relative to the PIA signal amplitude at one light intensity for (c) α-haematite and (d) anatase TiO2 photoanode.

Figure S6 shows J–V curves recorded under illumination intensities ranging from 0.4 to 3 suns. All curves display similar onset potentials and slopes, indicating that the applied potential predominantly sets the electron quasi-Fermi level. These results support our assertion that the observed kinetic regime

shift is driven by internal carrier dynamics (i.e., hole accumulation) rather than changes in applied potential. This interpretation is consistent with previous reports, [5] where J–V curves remained insensitive to transitions between different kinetic regimes. This further underlines the need for complementary operando spectroscopic techniques to detect mechanistic changes that are not visible in standard electrochemical measurements.

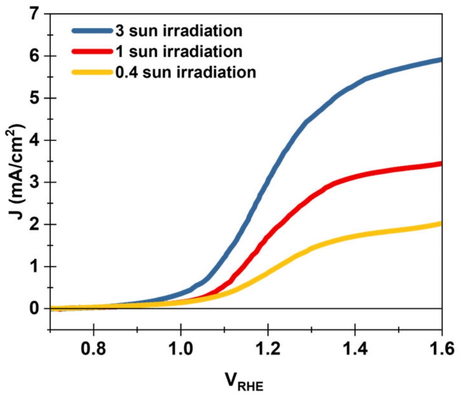

Figure S6 J–V curves of hematite photoanodes recorded under varying illumination intensities (0.4 to 3.0 suns) in 1 M NaOH (pH 14), scan rate: 20mV/s.

REFERENCES

(1) Righi, G.; Plescher, J.; Schmidt, F.-P.; Campen, R. K.; Fabris, S.; Knop-Gericke, A.; Schlögl, R.; Jones, T. E.; Teschner, D.; Piccinin, S. On the origin of multihole oxygen evolution in haematite photoanodes. *Nature Catalysis* **2022**. DOI: 10.1038/s41929-022-00845-9.

(2) Wang, D.; Sheng, T.; Chen, J.; Wang, H.-F.; Hu, P. Identifying the key obstacle in photocatalytic oxygen evolution on rutile TiO2. *Nature Catalysis* **2018**, *1* (4), 291-299. DOI: 10.1038/s41929-018-0055-z.

(3) Hankin, A.; Alexander, J. C.; Kelsall, G. H. Constraints to the flat band potential of hematite photo-electrodes. *Physical Chemistry Chemical Physics* **2014**, *16* (30), 16176-16186, 10.1039/C4CP00096J. DOI: 10.1039/C4CP00096J.

(4) Liu, T.; Wang, P.; Li, W.; Wang, D. Z.; Lekamge, D. D.; Chen, B.; Houle, F. A.; Waegele, M. M.; Wang, D. Temperature-Dependent Water Oxidation kinetics: implications and insights. *ACS Central Science* **2024**, *11* (1), 91-97.

(5) Bevan, K. H.; Peter, L. M. Do potential dependent kinetics play a role in photocatalytic rate trends? *Environmental Science: Nano* **2024**, *11* (2), 645-656.

(6) Kay, A.; Cesar, I.; Grätzel, M. New benchmark for water photooxidation by nanostructured α-Fe2O3 films. *Journal of the American Chemical Society* **2006**, *128* (49), 15714-15721.

(7) Kafizas, A.; Ma, Y.; Pastor, E.; Pendlebury, S. R.; Mesa, C.; Francàs, L.; Le Formal, F.; Noor, N.; Ling, M.; Sotelo-Vazquez, C. Water oxidation kinetics of accumulated holes on the surface of a TiO2 photoanode: a rate law analysis. *Acs Catalysis* **2017**, *7* (7), 4896-4903.

(8) Le Formal, F.; Pastor, E.; Tilley, S. D.; Mesa, C. A.; Pendlebury, S. R.; Grätzel, M.; Durrant, J. R. Rate law analysis of water oxidation on a hematite surface. *Journal of the American Chemical Society* **2015**, *137* (20), 6629-6637. Ma, Y.; Mesa, C. A.; Pastor, E.; Kafizas, A.; Francas, L.; Le Formal, F.; Pendlebury, S. R.; Durrant, J. R.

Rate law analysis of water oxidation and hole scavenging on a BiVO4 photoanode. *ACS Energy Letters* **2016**, *1* (3), 618-623.
